# Memory and motivational/emotional processes

**DOI:** 10.3389/fnbeh.2012.00071

**Published:** 2012-11-02

**Authors:** Antonella Gasbarri, Carlos Tomaz

**Affiliations:** ^1^Department of Applied Clinical and Biotechnologic Sciences, University of L'AquilaL'Aquila, Italy; ^2^Laboratory of Neurosciences and Behavior, Department of Physiological Sciences, Institute of Biology, University of BrasíliaBrasília, Brazil

As we know from our own experiences and the findings of many studies, emotional events are remembered with greater accuracy, vividness, and persistency compared to events lacking an emotional component (LaBar and Cabeza, 2006; Roozendaal and McGaugh, 2011). How emotional memory is controlled and regulated? This question has fascinated scientists and clinicians for a long time; in fact, the field focused on memory and motivational/emotional processes represents one of the fastest growing areas of neuroscience research. The selectivity that arousal creates is generally beneficial, as emotionally arousing situations in our lives are worth remembering, so that they can be savored and/or instructive. From an evolutionary point of view, it seems logical that a confrontation with an emotionally arousing event, such as a stressful one, is better remembered than a neutral one, resulting in a more adequate motivation to react in a similar situation.

Why emotional arousal enhances memory? Taking into account that neural processes initiated by an experience perseverate and consolidate over time, a possible explanation is that emotional arousal could activate neurobiological processes that modulate the consolidation of memories of recent experiences.

This special issue includes original papers and review articles that cover cutting-edge research in the interplay between memory, motivation, and emotion, providing the reader with what is up and coming with respect to research findings, theoretical advances, and methodological techniques. Many of the current “hot” topics in the field are covered, including the involvement of specific cerebral regions on the interaction between memory and motivational/emotional processes, the contribution of neurotransmitters and neuromodulators, and the role of arousal and stress.

The enhanced memory for emotional events has been attributed to the involvement and interaction of brain regions, in particular between the amygdala and other areas such as the hippocampal formation and prefrontal cortex (Phelps, 2004; Richter-Levin, 2004; McIntyre et al., 2012). The amygdala is active during emotional situations, and this activity influences the encoding and consolidation of the memory trace for the emotional event (McGaugh, 2004). On the light of previous evidence, some papers of this special issue focus on the role of specific neural regions in the interplay between memory and motivational/emotional processes, such as cortical and mesocorticolimbic areas (Martínez-Moreno et al., 2011; El Rawas et al., 2012; Holloway-Erickson et al., 2012; Puglisi-Allegra and Ventura, 2012), hippocampal formation (Hori et al., 2011; Garín-Aguilar et al., 2012), amygdala, substantia nigra, and striatum (Salado-Castillo et al., 2011; Wolf et al., 2011), septal nuclei (Matsuyama et al., 2011), nucleus accumbens (Núñez-Jaramillo et al., 2012), and autonomic nervous system (Garcia et al., 2011). Another group of papers analyzes the role and interaction of neurotransmitters and neuromodulators, such as catecholamines (Puglisi-Allegra and Ventura, 2012), endocannabinoids (Campolongo et al., 2012), acetylcholine and glucocorticoids (Fornari et al., 2012; Sánchez-Resendis et al., 2012) on memory and motivational/emotional processes. In order to highlight the impact of motivation and emotion on memory, functional neuroimaging techniques were used, including multichannel electroencephalography (EEG) (Arnone et al., 2011; Garcia et al., 2011; Uribe et al., 2011) and functional magnetic resonance imaging (fMRI) (Jepma et al., 2012; Rosales-Lagarde et al., 2012). Moreover, taking into account that recent studies have revealed seemingly large, but previously unsuspected, sex-related influences on the well-known mechanism that emotional events are better memorized than neutral events, this special issue includes evidence of sex-related differences in memory and talkativeness for emotional stimuli (Arnone et al., 2011). Finally, considering that in recent years a key conceptual issue, that warrants attention, is the fact that many studies examining emotional memory have focused on the highly arousing nature of emotional stimuli or experimental contexts, as the key component contributing to the enhancement of memory, some papers of this special issue discuss the involvement of arousal and stress in the interplay between memory, motivation, and emotion (Cruciani et al., 2011; Uribe et al., 2011; Packard and Goodman, 2012).

In conclusion, we hope that this special issue have provided evidence of the important and rapid progresses in this very interesting and relevant topic, and may give a significant contribution to the knowledge of how memory can be affected by emotional experiences, and related motivation. Then, taking into account that this emergent field is in continuous and fast growing, we strongly hope that the present special issue may *motivate* many neuroscientists to conduct other studies, paving the way for the next great theories and advances.
